# Attitudes, Willingness, and Barriers Among Hospital Pharmacists Toward Artificial Intelligence Integration in Pharmacy Practice: A Cross-Sectional Survey

**DOI:** 10.7759/cureus.89990

**Published:** 2025-08-13

**Authors:** Sara Shabana, Doua Alsaad, Lama Madi, Bhagyasree Sankar, Afif Ahmed, Wessam Elkassem, Moza Al Hail

**Affiliations:** 1 Pharmacy Department, Hamad Medical Corporation, Doha, QAT

**Keywords:** artificial intelligence, attitude, barriers, hospital pharmacist, pharmacy, willingness

## Abstract

Background

Artificial intelligence (AI) applications are increasingly being adopted in different healthcare sectors. Hospital pharmacists are an essential part of the multidisciplinary patient care team, and their perception of AI-driven technology is significant. This study aims to assess hospital pharmacists' perspectives on the use of AI in pharmacy practice, focusing on their attitudes, willingness, and perceived challenges.

Methods

An observational cross-sectional study was conducted using a web-based survey for hospital pharmacists working at Hamad Bin Khalifa Medical City (HBKMC), Qatar. The survey collected data on sociodemographics, willingness, attitudes, and barriers toward AI technology. The research questionnaire was developed and adopted based on an extensive literature review of similar studies, with minor changes to fit the current research setting. Descriptive data were reported. Chi-square (χ²) and Fisher’s Exact tests were used as appropriate. Statistical significance was set at p < 0.05.

Results

Ninety-three (66.4%) hospital pharmacists responded to the questionnaire. Around 59 (63.4%) of the participants were older than 35 years, with 66 (70.9%) having more than 5 years of experience in hospital settings. Participants demonstrated a positive willingness, with a median score of 50 (IQR: 42-53.5), and favorable attitudes, with a median score of 53 (IQR: 47-57), toward AI implementation in pharmacy practice. The highest willingness to adopt AI technology among respondents was in the area of enhancing patient adherence (79.6%, n = 47), while the strongest positive attitude reflected interest in staying updated on AI applications in pharmacy practice (89.2%, n = 83). The most commonly reported barriers were challenges in addressing patients’ emotional well-being and insufficient AI training, both noted by 75.3% (n = 70) of pharmacists. No significant association was detected between sociodemographic factors and hospital pharmacists’ willingness or attitudes toward AI integration (p-value > 0.05).

Conclusion

Generally, hospital pharmacists demonstrated a positive willingness and attitude toward applications of AI technologies, while emphasizing key challenges such as concerns about the loss of empathetic care and limited training. These findings underline the need for targeted educational programs to facilitate the AI digital transformation of the health organization. Future research to evaluate the long-standing effects of AI-related training and implementation on a broader institutional setting is recommended.

## Introduction

Artificial intelligence, referred to as AI, was first introduced in literature by AI researchers like Alan Turing and John McCarthy [1]. In 1997, McCarthy defined AI as “. . . the science and engineering of making intelligent machines, especially intelligent computer programs. It is related to the similar task of using computers to understand human intelligence, but AI does not have to confine itself to methods that are biologically observable.” [2]. This revolution in technology has reformed a variety of industries worldwide [3]. The introduction of AI in health systems, including the pharmacy sector, has significantly changed the way healthcare services are delivered [4-6]. AI technologies have transformed how pharmacists provide care by enhancing medication management and optimizing workflows. Machine learning (ML) applications allow pharmacists to dedicate more time to patient care activities, which can enhance clinical outcomes [7,8]. Furthermore, studies have shown that AI-powered automation tools optimize pharmacy inventory management, reduce errors, facilitate research, and contribute to education [9-11]. Ultimately, the adoption of AI is influencing the overall advancement of the healthcare sector and therapeutic outcomes.

Recently, research has assessed the perceptions, attitudes, and willingness of pharmacists toward artificial intelligence use in their practice [12,13]. One survey assessed community pharmacists’ opinions toward AI technology [14]. The results showed that pharmacists have positive willingness and attitudes toward AI technologies; however, different barriers were identified, including the lack of AI-related software and hardware, the need for human supervision, and the high cost related to AI implementation. Similarly, a questionnaire was conducted among pharmacy students and faculty, which highlighted pharmacists' knowledge, attitudes, and practices to adopt AI-driven applications in healthcare [15]. The study respondents' attitudes toward AI were optimistic; however, concerns regarding AI's impact on patient safety and pharmacy job security were pinpointed. Moreover, a survey focusing on the use of ChatGPT® (OpenAI, San Francisco, USA) only in pharmacy included various pharmacists, with hospital pharmacists comprising less than 38% of participants [16]. Research findings reported that despite favoring the use of ChatGPT in pharmacy practice, overall concerns about data accuracy, ethics, privacy, legal aspects, and bias risk were stated. 

Pharmacists and pharmacy students are becoming more aware of AI technologies, but incorporating them into everyday hospital practice remains a challenge. So far, very few studies have looked at how hospital pharmacists specifically feel about using AI, and none have focused on their views alone. 

Understanding hospital pharmacists’ perceptions and addressing their concerns toward the implementation of AI is crucial for the deployment of ML systems into their professional workflows. The current study aimed to exclusively assess hospital pharmacists’ attitudes and willingness toward AI integration, as well as to identify barriers to its application.

## Materials and methods

Study design

An observational cross-sectional study was conducted over three months using a web-based survey distributed via SurveyMonkey® (SurveyMonkey, Inc., San Mateo, USA from July to September 2024. The consent form was included, and respondents indicated their voluntary agreement to participate by completing the full survey. All information remained confidential and was used solely for research purposes.

Study population and setting

Hospital pharmacists working at Hamad Bin Khalifa Medical City (HBKMC) in Qatar were included in the study population. HBKMC consists of three specialized hospitals: Qatar Rehabilitation Institute (QRI), Ambulatory Care Center (ACC), and Women's Wellness and Research Center (WWRC). It is considered one of the largest hospital networks in Qatar, and it employs a high number of practicing licensed pharmacists.

Eligible pharmacists who matched the inclusion criteria were contacted via email. The study included all pharmacists employed by HBKMC, regardless of their age, gender, length of employment, professional role, or training background. Hospital pharmacists from diverse sections were included, such as clinical pharmacy, outpatient dispensing, inpatient services, narcotic control, intravenous (IV) admixture, drug information, and administrative pharmacy. Pharmacists involved in the preliminary pilot testing, along with pharmacy students and residents, were excluded.

Outcome measures

The primary outcome was to evaluate hospital pharmacists’ perceptions regarding AI adoption, measured through a validated questionnaire that assessed their willingness, attitudes, and perceived barriers. The secondary outcome was to examine the correlation between hospital pharmacists’ attitudes and willingness to AI implementation and their demographic characteristics. The outcome measures were derived from previous literature and expert opinion. 

For this study, attitude refers to perceptions and beliefs that pharmacists have toward the integration of AI in practice, willingness describes pharmacists' readiness, intention to adopt AI tools and technologies into practice, and barriers indicate obstacles or challenges that may hinder pharmacists from utilizing AI in their daily practice.

Questionnaire development and validation

The questionnaire was developed based on an extensive literature review and adapted from previously published studies with minor changes [14, 15, 17] to reflect the local hospital pharmacy settings. Revisions included language modifications and the removal of any items related to community pharmacy practice. The questions were culturally adapted to fit the population studied.

The survey was divided into four sections: sociodemographic (nine items), the willingness of participants toward AI implementation at hospital pharmacy (13 items), attitude of participants toward AI adoption in hospital pharmacy (14 items), and barriers to AI implementation (13 items). Based on these four sections, a web-based survey was developed using SurveyMonkey. For both willingness and attitude, scoring was performed based on a 5-point Likert scale, 1 point for strongly disagree and 5 points for strongly agree. The maximum possible score for willingness was 65, and for attitude was 70. Participants were grouped into high or low score groups based on whether they had a score above the median or not. For barriers, a dichotomous reporting scale was used to report whether each potential point was perceived as a challenge, facilitating rapid response and ease of analysis. For this research, AI is defined as any digital tool that uses automated decision-making support or machine learning (ML) associated with pharmacy services.

Content and face validity were ensured through a panel of three pharmacists with extensive knowledge in practice and research, as well as experience in creating survey tools. Furthermore, the survey was piloted among 10 hospital pharmacists to ensure the clarity of the questions, the estimated time to complete the survey, and the relevance of each item. Minor modifications were made to the survey instrument to eliminate ambiguous or leading questions and ensure alignment with the study's scope.

To assess internal consistency, Cronbach’s alpha was calculated for the main domains of the questionnaire, including willingness, attitudes, and perceived barriers. A reliability coefficient (α) of ≥0.70 was considered acceptable for internal reliability.

Different measures were implemented to minimize dropout rates, such as sending reminder emails and ensuring a user-friendly survey interface. In the survey questions, participants were stratified based on relevant variables (e.g., demographic characteristics) to ensure representativeness and enable robust subgroup analysis. The full questionnaire is provided in the Appendices (Appendix 1).

Data collection procedure

An anonymous, self-administered questionnaire accessible via a web link at SurveyMonkey was used to collect data. The hospitals' pharmacists from the three HBKMC specialized hospitals received the link via the hospital's official email system. A comprehensive description of the study was provided, including its objectives, data collection methods, measures for maintaining anonymity and confidentiality, and the voluntary nature of participation.

The rates of response were monitored, and a follow-up email was sent every two weeks to encourage participation in the survey until the desired sample size was reached.

Sample size calculation

The sample size was determined using Raosoft® sample size calculator (Raosoft Inc., Seattle, USA) [18]. Given a total targeted population of 140 hospital pharmacists working in HBKMC, a sample size of 90 was calculated, with a 5% margin of error, a 95% confidence level, and an 80% expected response distribution.

Data analysis

The data collected was analyzed using the statistical package SPSS version 22.0 (IBM Corp., Armonk, USA). The Shapiro-Wilk test was used to assess the normality of the continuous variable, and the result showed that the data were not normally distributed. For categorical data, descriptive analysis was used and presented as frequencies and percentages. In contrast, for numerical data, the median and 95% confidence interval were presented.

Based on the median of willingness and attitude scores, participants are divided into two groups: applicants with scores higher than the median were categorized in the "high" group, while those who scored lower were classified as "low" [19].

Factors associated with pharmacist willingness and attitude (age, gender, highest education level, and experience) were assessed by using the Chi-square (χ2) and Fisher's Exact tests as appropriate. P-values <0.05 were considered statistically significant.

Ethical approval

This study was approved by the Medical Research Center (MRC), Hamad Medical Corporation (approval no. MRC-01-24-215).

## Results

Demographic profile of hospital pharmacists

A total of 93 (66.4%) hospital pharmacists participated in the study, exceeding the targeted sample size. The age group for most of the pharmacists was between 35 and 44 years (n = 35, 38.7%), and between 25 and 34 years (n = 34, 36.5%). The participation of females was higher than males (n = 60, 64.5%). Most respondents (n = 46, 49.4%) held bachelor’s degrees only, while the majority of them had more than 5 years of experience in Qatar hospital practice (n = 66, 70.9%). A large number of the pharmacists (n = 79, 84.9%) reported familiarity with AI before this survey. A total of 30 (32.3%) of enrolled pharmacists rated their knowledge of AI in pharmacy practice as 3, which was the most frequent rating, on a scale of 1 (lowest) to 5 (highest). Similarly, the predominant rating for surveyed participants on the understanding of AI advantages was 3 (n = 32, 34.4%), while the most common rating for their understanding of AI disadvantages was 2 (n = 30, 32.3%). The sociodemographic characteristics of the study participants are shown in Table 1.

**Table 1:** Sociodemographic characteristics of the hospital pharmacists (N= 93)

Variable	Response Categories	N (%)
Age in Years	25 to 34	34 (36.56)
	35 to 44	36 (38.71)
	45 to 54	21 (22.58)
	55 to 64	2 (2.15)
Sex	Male	33 (35.48)
	Female	60 (64.52)
Highest Educational Level	Bachelor’s	46 (49.46)
	PharmD/Master’s	44 (47.31)
	PhD	3 (3.23)
Country of Bachelor’s Degree Certificate	Qatar	28 (30.11)
	Others	65 (69.89)
Years of Experience in Hospital Pharmacy in Qatar	<1 year	7 (7.53)
	1-5 years	20 (21.51)
	6-10 years	30 (32.26)
	11-15 years	18 (19.35)
	>15 years	18 (19.35)
Knowledge About AI Before Survey	Yes	79 (84.95)
	No	14 (15.05)
Knowledge of AI in Pharmacy Practice	1 (lowest)	14 (15.05)
	2	21 (22.58)
	3	30 (32.26)
	4	22 (23.66)
	5 (highest)	6 (6.45)
Understanding of AI Advantages	1 (lowest)	12 (12.90)
	2	17 (18.28)
	3	32 (34.41)
	4	25 (26.88)
	5 (highest)	7 (7.53)
Understanding of AI Disadvantages	1 (lowest)	12 (12.90)
	2	30 (32.26)
	3	28 (30.11)
	4	20 (21.51)
	5 (highest)	3 (3.23)

Hospital pharmacists’ willingness to integrate artificial intelligence into practice

Pharmacists showed a high willingness to implement AI technology, with a median score of 50 (42-53.5) out of a maximum possible score of 65. Participants’ highest willingness to apply AI technology (as indicated by 'agree' and 'strongly agree' statements) was for improving patient adherence (n = 74, 79.6%). On the other hand, the lowest willingness to utilize AI technology (as indicated by 'disagree' and 'strongly disagree' statements) was for patient counseling (n = 27, 29%). Other responses for pharmacists surveyed are presented in Table 2.

**Table 2:** Willingness of the pharmacists to apply AI technology (N=93) Participant responses (N=93) to survey items measuring pharmacists’ willingness to apply artificial intelligence (AI) technology in pharmacy practice. Responses were recorded using a 5-point Likert scale ranging from “Strongly Disagree” to “Strongly Agree.” Data are presented as frequency (%) for each response category. Median and 95% confidence intervals (CI) are reported where applicable. Willingness score is presented as median (interquartile range): 50 (42-53.5). “Less willingness” and “more willingness” categories were defined using the median score (50) and are presented as numbers (percentage).

Item	Frequency (%) or Median (95% Cl)				
	Strongly Disagree	Disagree	Neutral	Agree	Strongly Agree
1. Medical/social data collection	2 (2.2)	6 (6.5)	24 (25.8)	44 (47.3)	17 (18.3)
2. Detecting hidden and undiagnosed diseases	1 (1.1)	14 (15.1)	18 (19.4)	44 (47.3)	16 (17.2)
3. Identifying/ resolving drug-related problems	1 (1.1)	9 (9.7)	13 (14)	50(53.8)	20 (21.5)
4. Identifying/ resolving medication errors	1 (1.1)	9 (9.7)	13 (14)	48(51.6)	22 (23.7)
5. Identifying/ resolving adverse drug reactions	1 (1.1)	10 (10.8)	13 (14)	46(49.5)	23 (24.7)
6. Specifying treatment outcome	2 (2.2)	14 (15.1)	19 (20.4)	41 (44.1)	17 (18.3)
7. Evaluating different treatment options	1 (1.1)	7 (7.5)	16 (17.2)	51 (54.8)	18 (19.4)
8. Guide decision-making process in medical treatment	5 (5.4)	15 (16.1)	18 (19.4)	40 (43)	15 (16.1)
9. Follow up and monitor patients	2 (2.2)	14(15.1)	20(21.5)	40 (43)	17 (18.3)
10. Connecting healthcare provider systems together	1 (1.1)	8 (8.6)	11 (11.8)	45 (48.4)	28 (30.1)
11. Improving patient adherence	1 (1.1)	9 (9.7)	9 (9.7)	56 (60.2)	18 (19.4)
12. Medication dispensing	6 (6.5)	11 (11.8)	17 (18.3)	44 (47.3)	15 (16.1)
13. Patient counseling	12 (12.9)	15 (16.1)	19 (20.4)	33 (35.5)	14 (15.1)
Willingness score 50 (42-53.50), Low willingness (n = 48, 51.6%), More willingness (n = 45, 48.4%)					

Attitudes of hospital pharmacists toward AI adoption in pharmacy practice

Table 3 presents the attitude toward AI technology implementation in pharmacy practice. Hospital pharmacists reported a positive attitude toward adopting AI technology in pharmacy with a median score of 53 (47-57) out of a maximum possible score of 70. The highest attitude (agree and strongly agree statements) was for ‘I like to be up to date with AI applications in pharmacy settings’ and ‘I like to receive training in AI because it’s important to improve my career as a hospital pharmacist’, reaching 83 (89.2%) and 82 (88.2%), respectively. The least favorable attitude (disagree and strongly disagree statements) was toward ‘I fear that AI could replace my job as a pharmacist’, which was reported by 31 (33.3%) of participants. 

**Table 3:** Attitude toward AI technology implementation in pharmacy (N= 93) Participant responses (N = 93) to attitude-related survey items regarding the implementation of artificial intelligence (AI) in pharmacy practice. Items were rated on a 5-point Likert scale ranging from “Strongly Disagree” to “Strongly Agree.” Data are presented as frequency (%) for each category. Where applicable, median and 95% confidence intervals (CI) are reported. Attitude score is presented as median (interquartile range): 53 (47–57). “Less positive attitude” and “More positive attitude” categories were defined using the median score (53) and are presented as numbers (percentage).

Item	Frequency (%) or Median (95% Cl)				
	Strongly Disagree	Disagree	Neutral	Agree	Strongly Agree
I like to be up to date with AI applications in pharmacy settings	1 (1.1)	3 (3.2)	6 (6.5)	47 (50.5)	36 (38.7)
I like to receive training in AI because it’s important to improve my career as a hospital pharmacist	2 (2.2)	4 (4.3)	5 (5.4)	42 (45.2)	40 (43)
I believe that AI will improve the services provided in the hospital pharmacy	1 (1.1)	5 (5.4)	11 (11.8)	44 (47.3)	32 (34.4)
I feel that AI will improve hospital pharmacy patient satisfaction	1 (1.1)	8 (8.6)	11 (11.8)	42 (45.2)	31 (33.3)
I fear that AI could replace my job as a pharmacist	7 (7.5)	24 (25.8)	19 (20.4)	32 (34.4)	11 (11.8)
I believe that AI applications will be widely used in pharmacy practice	1 (1.1)	8 (8.6)	18 (19.4)	45 (48.4)	21 (22.6)
I believe that AI can help reduce medication errors	1 (1.1)	8 (8.6)	13 (14)	48 (51.6)	23 (24.7)
I feel that AI will enhance the process of decision-making in medical treatment	2 (2.2)	10 (10.8)	19 (20.4)	44 (47.3)	18 (19.4)
I feel that AI will assist in performing clinical investigation tasks more efficiently than humans	4 (4.3)	17 (18.3)	19 (20.4)	40 (43)	13 (14)
I feel that AI can enhance the pharmacist’s role in patient follow-up and monitoring	2 (2.2)	5 (5.4)	22 (23.7)	49 (52.7)	15 (16.1)
I believe that AI always makes the best choice since it never gets tired (mentally or physically)	7 (7.5)	21 (22.6)	22 (23.7)	28 (30.1)	15 (16.1)
I believe that AI can multitask more effectively than humans and analyze data more quickly	1 (1.1)	6 (6.5)	21 (22.6)	45 (48.4)	20 (21.5)
I feel that AI can improve patient outcomes	1 (1.1)	7 (7.5)	22 (23.7)	49 (52.7)	14 (15.1)
I feel that AI can reduce the cost of care	7 (7.5)	14 (15.1)	17 (18.3)	43 (46.2)	12 (12.9)
Attitude score 53 (47-57), Less positive attitude (n = 50, 53.8%), More positive attitude (n = 43, 46.2%)					

Perceived barriers to AI implementation among hospital pharmacists

The most frequently identified barriers for implementing AI in hospital pharmacy were ‘the challenge of empathizing and considering the patient's emotional well-being’ and ‘lack of AI training’, where each barrier was reported by 70 (75.3%) of participants. Other detected limitations included ‘AI requires human supervision’, ‘If a vital decision (such as end-of-life care) must be taken, the AI cannot make the decision and will generate an unreliable report’, and ‘Regulatory and social limitations may create challenges that restrict how effectively AI can support medical practitioners’, which were reported by 68 (73.1%), 67 (72%), and 65 (69.9%) participants, respectively. On the other hand, ‘Difficulty to apply AI’ and ‘Lack of time’ were less frequently identified by respondents as barriers to adopting AI technology in hospital pharmacy at 42 (45.2%) and 44 (47.3%) participants, respectively. Other barriers are shown in Table 4.

**Table 4:** Barriers to implement AI technology in the hospital pharmacy (N=93) Summary of hospital pharmacists’ perceived barriers to implementing artificial intelligence (AI) technology in practice (N=93). The questionnaire items were assessed using a dichotomous scale: “Agree” or “Disagree”. Data are presented as frequency (%) for each response category. Where relevant, median and 95% confidence intervals (CI) are reported.

Item	Frequency (%)	
	Disagree	Agree
1. Lack of AI Information	30 (32.3)	63 (67.7)
2. Lack of AI flexibility	34 (36.6)	59 (63.4)
3. The challenge of empathizing and considering the patient's emotional well-being	23 (24.7)	70 (75.3)
4. Difficulty to apply AI	51 (54.8)	42 (45.2)
5. Lack of AI training	23 (24.7)	70 (75.3)
6. High running cost of AI	37 (39.8)	56 (60.2)
7. Concerns about legal action	41 (44.1)	52 (55.9)
8. Lack of time	49 (52.7)	44 (47.3)
9. Translating medical terminology into machine language involves collaboration between healthcare practitioners and AI	36 (38.7)	57 (61.3)
10. AI requires human supervision	25 (26.9)	68 (73.1)
11. If a vital decision (such as end-of-life care) must be taken, the AI cannot make the decision and will generate an unreliable report	26 (28)	67 (72)
12. Regulatory and social limitations may create challenges that restrict how effectively AI can support medical practitioners	28 (30.1)	65 (69.9)
13. Lack of AI-related software and hardware	35 (37.6)	58 (62.4)

Demographic associations with willingness and attitudes

As shown in Table 5, none of the tested sociodemographic characteristics were associated with higher levels of willingness to implement AI technology in hospital pharmacy (p-value > 0.05). Similarly, age, gender, education level, and experience were not associated with pharmacists’ attitudes toward utilizing AI applications as represented in Table 6 (p-value > 0.05).

**Table 5:** Factors associated with pharmacists’ willingness toward implementing AI technology Association between selected demographic and professional variables and pharmacists’ willingness, categorized as “Less Willingness” and “More Willingness” based on survey scores. Willingness was assessed using a 5-point Likert scale (1 = Strongly Disagree to 5 = Strongly Agree), with a maximum possible score of 65. Participants scoring above the median (50) were classified as “More Willingness,” while those scoring at or below were classified as “Less Willingness.” Chi-square (χ²) and Fisher’s Exact tests were used as appropriate. Statistical significance was set at p < 0.05.

Variable		Less Willingness (N (%))	More Willingness (N (%))	P-Value
Age in Years	25 to 34	33 (37.9)	1 (16.7)	0.389
	35 to 44	34 (39.1)	2 (33.3)	
	45 to 54	18 (20.7)	3 (50.0)	
	55 to 64	2 (2.3)	0	
Gender	Female	57 (65.5)	3 (50.0)	0.442
	Male	30 (34.5)	3 (50.0)	
Highest education level	Bachelor’s	42 (48.3)	4 (66.7)	-
	PharmD/Master’s	43 (49.4)	1 (16.7)	
	PhD	46 (49.5)	44 (47.3)	
Experience in Years	<1	7 (8.0)	0	0.287
	>15	17 (19.5)	1 (16.7)	
	1-5	20 (23.0)	0	
	11-15	15 (17.2)	3 (50)	
	6-10	28 (32.2)	2 (33.3)	

**Table 6:** Factors associated with pharmacists’ attitude toward implementing AI technology Association between selected variables and pharmacist attitudes, categorized as “Less Positive Attitude” and “More Positive Attitude” based on total attitude scores. Attitude was assessed using a 5-point Likert scale (1 = Strongly Disagree to 5 = Strongly Agree), with a maximum possible score of 70. Participants scoring above the median (53) were classified as having a “More Positive Attitude,” and those scoring at or below were classified as “Less Positive Attitude.” Chi-square (χ²) and Fisher’s Exact tests were used as appropriate. Statistical significance was set at p < 0.05.

Variable		Less positive attitude (N (%))	More positive attitude (N (%))	P-value
Age in Years	<45	65 (92.9)	5 (7.1)	0.807
	>45	21 (91.3)	2 (8.7)	
Gender	Female	54(90)	6 (10)	0.223
	Male	32 (97)	1 (3)	
Highest education level	Bachelor’s	44 (95.7)	2 (4.3)	0.398
	PharmD/Master’s	39 (88.6)	5 (11.4)	
	PhD	3 (100)	0 (0)	
Experience in Years	=5	25 (92.6)	2 (7.4)	0.978
	>5	61 (92.4)	5 (7.6)	

## Discussion

Key findings

The integration of AI into medical practices, particularly within hospital facilities, has attracted significant attention from healthcare practitioners. The current study uniquely assessed hospital pharmacists' perspectives on AI adaptation into pharmacy practice. The survey focused on attitudes, willingness, and the challenges they may face due to the application of AI in pharmacy. The results overall were positive for pharmacist attitude and willingness to utilize AI-driven technologies. Still, several barriers have been reported, including a lack of AI-directed training and the inability of AI technology to achieve empathetic engagement. 

In this study, respondents demonstrated a positive willingness to apply AI tools to their hospital practice, which is consistent with past literature reporting high levels of acceptance [12,14]. Moreover, aligning with the literature, the majority of participants believe that AI can reduce medication errors and detect drug-related problems (DRPs) [17]. These findings emphasize the change in culture around the utilization of AI, which can play a significant role in advancing the healthcare sector revolution.

In our study, hospital pharmacists indicated a favorable attitude toward the implementation of AI into pharmacy practice, aligning with previously published studies [12-14]. Interestingly, contrary to reported findings, only 43 (46.2%) of the hospital pharmacists in this survey reported concerns about AI taking over their jobs. Other studies have reported significant concerns regarding AI's ability to replace human expertise [20-23]. This reflects that our respondents believe their profession as hospital pharmacists will not become redundant due to AI. Nonetheless, in agreement with similar research that assessed clinicians’ perspectives on AI adaptation, our study revealed that 82 (88.2%) of respondents agreed that AI-related training is needed [12,14,21].

Our study showed that many participants believe pharmaceutical care applications can be enhanced with AI integration compared to traditional practice. These results are in alignment with existing literature showing that AI-driven automation of routine tasks can enable pharmacists to focus more on direct patient care [24,25]. Innovative AI tools can reveal overlooked medical information in large databases, which can help reduce medication errors and reduce workload [6,26,27]. Nonetheless, 76 (81.7%) of respondent in our study believe that AI can improve services provided in hospital pharmacy, highlighting their enthusiasm and showing the need for targeted AI training programs to support future hospital utilization of AI.

While previous studies have reported an association between sociodemographic factors and pharmacist attitudes and willingness to adopt AI technologies, this study did not find any significant relation [12,14]. The lack of correlation in this study may be attributable to different practice settings, character differences, or greater knowledge of AI utilization. Moreover, due to earlier research linking demographic factors and perceptions of AI, future studies involving larger and more diverse respondents may be needed to learn more about factors affecting perceptions of AI in hospital pharmacists.

This research showcases the barriers identified by hospital pharmacists toward deploying AI tools in the pharmacy field. Consistent with the literature, two of the major barriers identified include a lack of AI training and AI reliance on human supervision [12,14]. However, communication with patients and showing empathy emerged as an underreported barrier in previous studies, suggesting respondents' concerns regarding the neglected emotional aspects of AI that may negatively affect patient care. As the majority of reported literature is based on community pharmacists' or pharmacy students’ perspectives [14-16], diversity in identified barriers is anticipated due to different practice settings. Ensuring AI training, along with the development of ethical frameworks, is crucial for effective AI implementation in hospital pharmacies.

Globally, ethical and legal aspects have been the major reported barriers among health care providers, especially physicians [28]. As dehumanization of care due to AI involvement falls under ethical considerations, our study findings mirror part of the concerns reported in the literature. As hospital-based pharmacists working in direct patient-care settings such as dispensing and clinical pharmacy services, who experience daily patient interactions, they acknowledge the inability of AI to deliver the same level of empathic engagement.

In our study, the majority of respondents were aged 35 years or older (n = 59, 63.4%), which reflects a gap in their academic exposure to AI principles, in contrast to the younger population, who are more likely to have encountered AI-focused courses [17]. Therefore, this may be associated with their perception of AI technology and its barriers. In Qatar, our study findings build upon a previous survey conducted within the Hamad Medical Corporation (HMC) that explored the understanding of all hospital workers regarding the use of AI [29]. Unlike our findings that uniquely reported the hospital pharmacist perspective, this study reflected the general views of different healthcare providers on AI. The current research data provides a more in-depth examination of pharmacy-related factors affecting their views and perceived challenges.

Strengths and weaknesses

There is a limited number of studies conducted to explore the perception of hospital pharmacists for AI integration into pharmacy practice, which makes the present study valuable by contributing to the existing body of literature and addressing the gap in current research. The study also has an adequate response rate that achieved the targeted sample size. Furthermore, the survey was distributed in a multi-specialty setting, as it included different hospitals in the State of Qatar. Also, the study included participants of all hospital pharmacy sections, including clinical, outpatient dispensing, inpatient, narcotic, intravenous (IV), drug information, and administrative pharmacy. The study was limited by its inclusion of a single hospital network, which may reduce the generalizability of the results. Additionally, the potential for response bias is higher due to survey data relying on self-reporting, as pharmacists with favorable views toward AI may be more likely to participate. Finally, this study did not compare a specific AI tool being used, which could have provided a better insight into pharmacists' interactions with AI in real-world settings.

Implications for practice and research

The current findings provide a tool for planning future AI implementation in the pharmacy field. Benchmarking hospital pharmacists’ views on barriers related to AI application is important, as it can facilitate focused training based on identified challenges, thus enhancing the pre-planning for AI models in hospitals. Therefore, we recommend that future research address operational and ethical concerns reported by pharmacists to shape their roles and propose action frameworks. Additionally, we encourage healthcare authorities and policymakers to develop AI-specific training for healthcare workforces, including pharmacists, before any AI adoption. Furthermore, a pilot implementation study or interventional research may be recommended to evaluate pharmacist-AI interactions with real-world tools.

## Conclusions

Overall, hospital pharmacists showed a positive willingness and attitude toward AI implementation in pharmacy practice. Concerns regarding empathetic patient interactions and the lack of AI-specific training emerged as key barriers. These findings highlight a strong level of awareness regarding the potential applications and benefits of AI in the pharmacy field. This research illustrates the need for AI educational initiatives to enhance pharmacists' digital literacy and foster confidence in AI use. Future studies should explore the long-term effects of AI-related education and implementation on larger institutional or regional settings.

## Appendices

Appendix 1: survey instrument

Survey Title: Attitude, Willingness, and Barriers of Hospital Pharmacists Towards Artificial Intelligence Integration in Pharmacy Practice

Study Reference:

Research MRC No: MRC-01-24-215 Conducted by: Pharmacy Department, Hamad Bin Khalifa Medical City (HBKMC) Contact: Sshabana@hamad.qa

Participant Invitation:

Dear colleague,

On behalf of the Pharmacy Department at HBKMC, we request your valuable participation in a research project exploring pharmacists' readiness for artificial intelligence (AI) integration in pharmacy practice. This includes your attitude, willingness, and perceived barriers.

The survey is anonymous and will take approximately 10 minutes to complete. Your input will contribute to strategic planning for future AI adoption within the hospital setting.

Thank you for your time and valuable contribution.

- Research Team

Section 1: Sociodemographic Information

**Table 7:** Section 1: Sociodemographic characteristics

Question	Response Options
Age	☐ 25–34 ☐ 35–44 ☐ 45–54 ☐ 55–64
Sex	☐ Male ☐ Female
Highest Educational Level	☐ Bachelor’s ☐ PharmD/Master’s ☐ PhD
Country of Bachelor’s Degree	☐ Qatar ☐ Others: __________
Years of Experience in Hospital Pharmacy in Qatar	☐ <1 ☐ 1–5 ☐ 6–10 ☐ 11–15 ☐ >15
Have you heard the term Artificial Intelligence (AI) before this survey?	☐ Yes ☐ No
How do you rate your knowledge about AI applications/machine learning? *(1 = lowest, 5 = highest)*	☐ 1 ☐ 2 ☐ 3 ☐ 4 ☐ 5
How do you rate your understanding of AI advantages?	☐ 1 ☐ 2 ☐ 3 ☐ 4 ☐ 5
How do you rate your understanding of AI disadvantages?	☐ 1 ☐ 2 ☐ 3 ☐ 4 ☐ 5

Section 2: Questions 1 to 13 assess your willingness to apply artificial intelligence (AI) technology in the hospital pharmacy:

**Table 8:** Questions 1 to 13 assess your willingness to apply artificial intelligence (AI) technology in the hospital pharmacy:

	Strongly agree	Agree	Neutral	Disagree	Strongly disagree
Medical/social data collection					
Detecting hidden and undiagnosed diseases					
Identifying/ resolving drug-related problem					
Identifying/ resolving medication errors					
Identifying/ resolving adverse drug reaction					
Specifying treatment outcome					
Evaluating different treatment options					
Guide decision making process in medical treatment					
Follow up and monitoring patients					
Connecting healthcare provider systems together					
Improving patient adherence					
Medication dispensing					
Patient counseling					

Section 3: Attitude Toward AI Implementation

Questions 1 to 14 are related to your attitude toward artificial intelligence (AI) technology implementation in the hospital pharmacy:

**Table 9:** Questions 1 to 14 are related to your attitude toward artificial intelligence (AI) technology implementation in the hospital pharmacy:

	Strongly agree	Agree	Neutral	Disagree	Strongly disagree
I like to be up-to-date in AI application in pharmacy setting					
I like to receive training on AI because it’s important to improve my career as a hospital pharmacist					
I believe that AI will improve the services provided in the hospital pharmacy					
I feel that AI will improve the hospital pharmacy patient satisfaction					
I fear that AI could replace my job as pharmacist					
I believe that AI applications will be widely used in pharmacy practice					
I believe that AI can help reduce medication errors					
I feel that AI will enhance process of decision making in medical treatment					
I feel that AI will assist in performing clinical investigation tasks more efficiently than humans					
I feel that AI can enhance pharmacist’s role in patient follow-up and monitoring					
I believe that AI always makes the best choice since it never gets tired (mentally or physically)					
I believe that AI can multitask more effectively than humans and analyze data more quickly					
I feel that AI can improve patient outcomes					
I feel that AI can reduce the cost of care					

Section 4: Barriers to Implementing AI

Questions 1 to 13 are related to barriers to implementing artificial intelligence (AI) technology in the hospital pharmacy:

**Table 10:** Questions 1 to 13 are related to barriers to implement artificial intelligence (AI) technology in the hospital pharmacy:

	Agree	Not sure	Disagree
Lack of AI Information			
Lack of AI flexibility			
The low ability to sympathize and consider the emotional well-being of the patient			
Difficulty to apply AI			
Lack of training			
High running cost of AI			
Fear of litigation			
Lack of time			
Translating medical terminology into machine language involves collaboration between healthcare practitioners and AI			
AI requires human supervision			
If a vital decision (such as end-of-life care) must be taken, the AI cannot make the decision and will generate an unreliable report			
Regulatory and social constraints may limit AI’s potential to help medical practitioners			
Lack of AI-related software and hardware			
